# Unveiling the potential of MXene-fabricated catalysts: an effective approach for H_2_ generation from water splitting†

**DOI:** 10.1039/d4na00754a

**Published:** 2024-10-16

**Authors:** Muhammad Zeeshan Abid, Khezina Rafiq, Abdul Rauf, Ejaz Hussain

**Affiliations:** a Institute of Chemistry, Inorganic Materials Laboratory 52S, The Islamia University of Bahawalpur 63100 Pakistan ejaz.hussain@iub.edu.pk khezina.rafiq@iub.edu.pk

## Abstract

Hydrogen has enough potential and can be successfully used as an alternative to the conventional fuel. It can be successfully produced from water that is not only a sustainable source but exists everywhere on earth. Additionally, its combustion releases water that is quite safe and environment friendly. The current project was designed to generate hydrogen from catalytic water splitting on TiO_2_@Ti_3_C_2_T_*x*_ catalysts. To obtain the required catalytic characteristics, titania was engineered on Ti_3_C_2_T_*x*_ surfaces *in situ* using an ethanol-assisted solvothermal approach. After careful recovery, the catalysts were characterized and assessed for the photoreaction. All photoreactions were performed in a quartz reactor (150 mL), where hydrogen evolution activities were monitored on GC-TCD (Shimadzu-JP). The comparative activities indicated that TiO_2_@C and TiO_2_@Ti_3_C_2_T_*x*_ catalysts deliver 9.37 and 18.57 mmol g^−1^ h^−1^ of hydrogen, respectively. The higher activities of TiO_2_@Ti_3_C_2_T_*x*_ were attributed to the existence of higher active sites (charge trapping centres) on the multilayer MXene that progressively promote and facilitate redox reactions. Reason is that existence of titania on MXene interfaces develops heterojunctions that rectify the charge transfer; hence reduce the charge recombination (*i.e.*, back reaction). On the basis of encouraging activities, it has been concluded that the aforementioned approach holds promise to replace the costly and conventional hydrogen generation technologies.

## Abstract 2

Ethanol assisted solvothermal approach was introduced to produce TiO_2_@Ti_3_C_2_T_*x*_ and TiO_2_@C catalysts for comparative H_2_ generation from water splitting.

## Graphical abstract

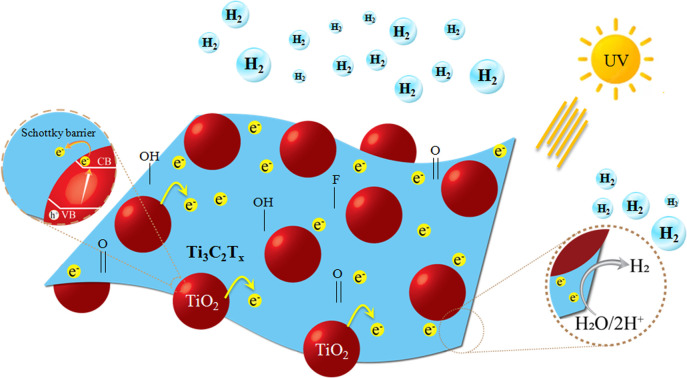

## Introduction

Without doubt, fossil fuels are the main sources of the world's energy, but pollution generated by their burning is enough to ruin life on earth. If the current speed of fossil fuel consumption continues, it will definitely bring a severe change in the earth's climate.^1^ Unpredictable weather, sudden hailstorms and rainfalls are undeniable evidence of climate change. Thus, for life on earth, an alternative and green source is urgently needed.^2^ That is why the scientific community is focusing on new and sustainable sources that not only meet the energy demand but also protect our earth and atmosphere. Additionally, excessive growth in the human population and industrialization increase these challenges. Due to high costs of conventional energy sources, developing countries are struggling to shift from fossil fuels to green and sustainable sources.^3^

Hydrogen is an ideal fuel due to its (i) high calorific value (150 kJ g^−1^), (ii) zero carbon emissions, and (iii) renewable nature.^4^ Water is a stable molecule, and it needs 237 kJ mol^−1^ of energy to split into hydrogen and oxygen. However, the activation energy of the water splitting reaction can be reduced with the use of stable and effective catalysts.^5^ For water splitting, commonly used photocatalysts are TiO_2_, ZrO_2_, ZnO, SrTiO_3_, BaTiO_3_, ZnS, CeVO_4_, CuO, CdS, CdZnS, g-C_3_N_4,_ ZnIn_2_S_4_, and Zn_3_V_2_O_8_.^6–14^ However, these catalysts have been restricted due to the following drawbacks: (i) fast charge recombination; (ii) poor optical response; (iii) photocorrosion; (iv) stability and durability; and (v) improper band gaps and structural defects.^15^ Due to the aforementioned drawbacks, these catalysts cannot split water efficiently; therefore, for progressive water splitting, improvements in the catalytic system have become necessary. Thus, modifications using co-catalysts like MXene or graphene have gained more interest due to their excellent conductivity.^16^

In recent decades, MXene (Ti_3_C_2_T_*x*_) has opened new pathways for developing stable and durable catalysts. The reason is that MXenes naturally exhibit surface terminations like –F, =O, and –OH that enhance the surface reaction ability of catalysts.^17^ Among the MXenes family, titanium carbide (Ti_3_C_2_T_*x*_) has gained significant attention for energy conversion and storage applications. Due to tuneable characteristics, increased conductivity, and intrinsic features, it facilitates more active sites during photoreactions.^18^

Among co-catalysts, MXene and graphene have been identified as exceptional co-catalysts that can effectively promote surface charges to the active centres (*i.e.*, redox sites). Additionally, these co-catalysts have substantial selectivity and can only perform in optimized catalytic reactions. However, the fabrication of MXene with TiO_2_ for targeted applications (*i.e.*, water splitting) is quite challenging having relatively high mechanical strength.^19^ Because the dispersion of MXene matrix on semiconductor's surfaces can be problematic, this causes agglomeration during the developmental process. Hence, the synthesis of TiO_2_/Ti_3_C_2_T_*x*_ and TiO_2_/C has been found significant factor for excellent photocatalytic activities.^20^ The advantage of MXene support is that it can become the inherent source of TiO_2_ growth due to Ti–C–Ti–C–Ti structural arrangements.^21^ Additionally, the complete oxidation of Ti_3_C_2_T_*x*_ produces the carbide-derived carbon (CDC), which also acts as co-catalyst and promotes the photon absorption ability of semiconductor systems.^22^ Thus, *in situ* developments of metal oxide, *i.e.*, TiO_2_, on Ti_3_C_2_T_*x*_ and CDC are quite important to enhance the overall catalytic efficiency of the photoreaction.

In this work, we have extended the utilization of MXene using *in situ*-engineered TiO_2_ (*i.e.*, TiO_2_@Ti_3_C_2_T_*x*_ and TiO_2_@C) *via* an ethanol-assisted solvothermal approach. The significant advantage of *in situ* growth of TiO_2_ has the following advantages for catalytic water splitting: (i) superior conductivity and charge transportation; (ii) formation of heterojunctions to reduce back reactions; (iii) extension of the internal optical response during photoreaction; (iv) enhanced stability and durability; (v) enhanced pool of reactive charges on the redox sites. The findings of the current project deliver encouraging examples of renewable energy systems that can be further be improved for large-scale implementation.

## Experimental

The details of the chemicals and instruments used in current work have been discussed in the ESI† section.

### Catalysts preparation

**Fig. 1:**
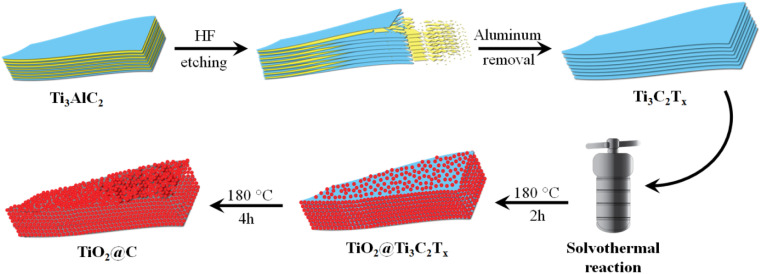
Graphical illustration for the synthesis of Ti_3_C_2_T_*x*_, TiO_2_@Ti_3_C_2_T_*x*_ and TiO_2_@C catalysts.

The scheme that was employed to design the MXene and derived catalysts is illustrated in Fig. 1. In this study, Ti_3_C_2_T_*x*_, *i.e.*, titanium carbide, has been developed by the etching process, which was designed and selectively performed with HF (*ca.* 40%) [20]. Typically, 500 mg of Ti_3_AlC_2_ was transferred to 40 mL of the aforementioned HF in a 100 mL Teflon reactor. Note that the reaction process was completed in a safe environment, *i.e.*, under a fume hood. To ensure the complete removal of aluminium, the product was vigorously stirred under gentle sonication [21]. The chemical reaction involved in this process is given in eqn (1).

1 Ti_3_AlC_2_ + 6HF → AlF_3_ + Ti_3_C_2_T_*x*_ + 3H_2_

To isolate Ti_3_C_2_T_*x*_ from the HF solution, the suspension underwent some important steps: (i) centrifugation at 5000 rpm; (ii) careful washing six times to eliminate impurities; and (iii) vacuum-drying at 85 °C.

In this study, TiO_2_@Ti_3_C_2_T_*x*_ and TiO_2_@C catalysts were synthesized *via* an ethanol-assisted solvothermal approach.^23^ The Ti_3_C_2_T_*x*_ was used as a precursor material because it has an inherent titanium source for the growth of TiO_2_ due to Ti–C–Ti–C–Ti structural arrangements.^24^ Typically, 200 mg of the aforementioned precursor support was dispersed in 40 mL of mixture solvents (with 80 : 20 water/ethanol ratios) and sonicated for 10 min. For the *in situ* growth of TiO_2_ on Ti_3_C_2_T_*x*_, this mixture was transferred to a Teflon-lined autoclave reactor for 2 h at 180 °C. The reaction was continued for a further 4 h to achieve the complete oxidation of MXene so that it is successfully transformed into the high-textured TiO_2_@C product. It has been noted that optimized conditions are necessary to obtain the complete oxidation of MXene for developing high-quality TiO_2_ catalyst textures *in situ* on the surfaces of carbide.^25^ After hydrothermal reaction, catalysts were recovered by vacuum filtration. To remove the impurities or unreacted precursors, catalysts were thoroughly rinsed with the ethanol/acetone mixture (80 : 20 v/v ratios). To eliminate the moisture from layered structures, catalysts were vacuum dried at 95 °C for 5 h.

### Photocatalytic activities

Photocatalytic water splitting reactions for hydrogen generation were carried out using a PLS-SXE300 lamp. The photoreactions were performed in a 150 mL quartz reactor, where a 20 mg dose of catalysts was optimised for 50 mL of pure water. H_2_ and O_2_ generation rates were quantified by taking gas samples (0.5 mL per syringe) from the headspace of the photoreactor. For the accurate analysis, high-purity argon served as the carrier gas. A specialized molecular sieve capillary column was used for gas separation. The gases were quantified using the internal calibration curves of the gas chromatography system (GC-TCD, Shimadzu, 2010).^26^ Quantum efficiencies (QEs) were calculated using eqn (2). Note that these measurements were executed under controlled conditions.2
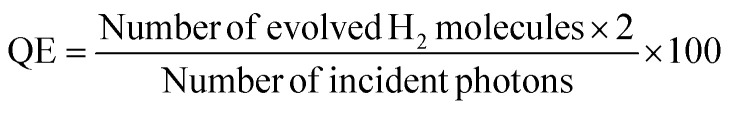


## Results and discussions

The synthesis scheme of Ti_3_C_2_T_*x*_, TiO_2_@Ti_3_C_2_T_*x*_, and TiO_2_@C catalysts is shown in Fig. 1. Various characterization techniques have been employed to study the structural, morphological, optical, magnetic, and chemical properties of the synthesized catalysts, which have been discussed below.

### XRD analyses

**Fig. 2:**
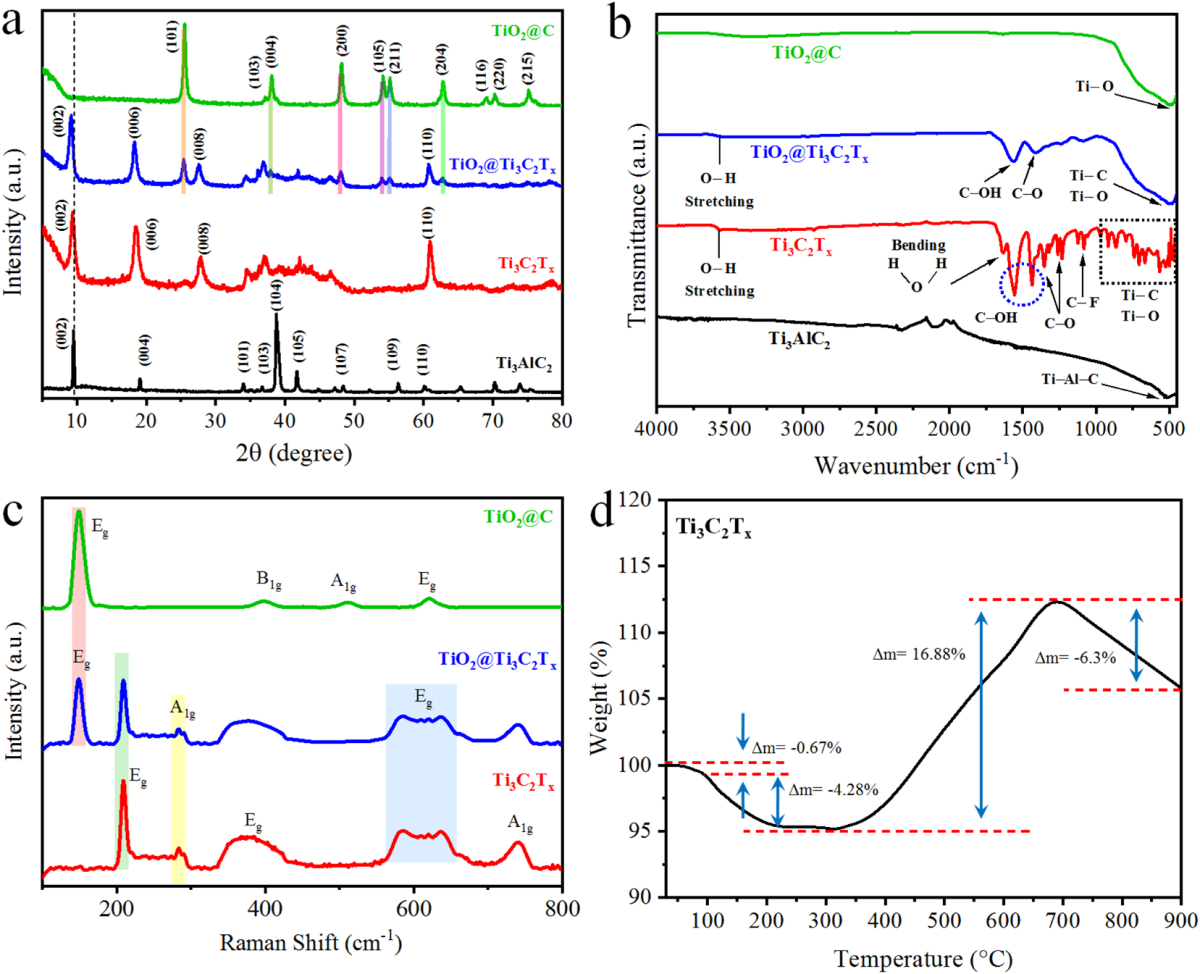
(a) XRD pattern; (b) FTIR results of Ti_3_AlC_2_, Ti_3_C_2_T_*x*_, TiO_2_@Ti_3_C_2_T_*x*_, and TiO_2_@C; (c) Raman spectroscopy results of Ti_3_C_2_T_*x*_, TiO_2_@Ti_3_C_2_T_*x*_, and TiO_2_@C; (d) TGA of Ti_3_C_2_T_*x*_.

The XRD patterns of Ti_3_AlC_2_ (MAX), Ti_3_C_2_T_*x*_ (MXene), TiO_2_@Ti_3_C_2_T_*x*_ and TiO_2_@C are described in Fig. 2a. XRD is an excellent tool that can accurately provide significant insights about structural transformations.^27^ Results of this study indicate that the XRD pattern of pristine Ti_3_AlC_2_ closely matches JCPDS No. 52-0875, validating its purity with hexagonal crystalline structure.^28^ Notably, the XRD patterns of Ti_3_C_2_T_*x*_ display broader lattice planes (002) as compared to Ti_3_AlC_2_. It is worth mentioning that shift of the (002) planes towards smaller angles along with the disappearance of the characteristic peak at 39° (*i.e.*, corresponds to (104) planes) indicate the complete removal of aluminium atoms (in the form of AlF_3_).^29^ These results confirm the phase purity and successful transformation of MAX to Ti_3_C_2_T_*x*_ structures. The TiO_2_@Ti_3_C_2_T_*x*_ catalysts exhibit the inherent diffractions of TiO_2_ (anatase) along with the Ti_3_C_*x*_T_*x*_ XRD patterns. The changes in diffraction pattern confirm the *in situ* growth of TiO_2_ over Ti_3_C_2_T_*x*_ surfaces. On the other hand, the peak positions of TiO_2_@C confirm the anatase structure and are exactly matched with PDF#73-1764.^30^ Additionally, removal of Ti_3_C_2_T_*x*_ diffraction peaks confirms the complete conversion (*i.e.*, oxidation) of Ti–C–Ti–C–Ti to the TiO_2_@C.^31^ XRD pattern and their corresponding *hkl* values for as reported catalysts have been tabulated in Table S1.† The structural changes observed in XRD results provide valuable insights into catalyst performance.

### FTIR analyses

The FTIR analysis (Fig. 2b) of Ti_3_AlC_2_ revealed the bending vibrations of the Ti–Al–C bond at 505 cm^−1^. The etching of aluminium was confirmed by the removal of the 505 cm^−1^ peak, whereas new vibration patterns are attributed to surface termination (T_*x*_ =O, –F, –OH).^32^ FTIR results indicate that the vibrations observed at 566 cm^−1^, 706 cm^−1^, 855 cm^−1^, and 922 cm^−1^ are attributed to the Ti–C/Ti–O stretching vibrations. The O-terminations on the Ti_3_C_2_T_*x*_ surface caused Ti–O vibrations at 617 cm^−1^. F-terminations are observed at 1057 and 1097 cm^−1^, whereas O–H terminations are detected at 1399 and 1437 cm^−1^.^33^ The vibrations at 1229 and 1343 cm^−1^ are attributed to adsorb/atmospheric CO_2_.^34^ These results confirm the formation of a multilayer Ti_3_C_2_T_*x*_ structure. In the FTIR results of TiO_2_@Ti_3_C_2_T_*x*_ catalysts, the strong vibration at 480 cm^−1^ confirms the formation of TiO_2_ over Ti_3_C_2_T_*x*_. The aforementioned results also demonstrate the surface terminations (=O and –OH) along with Ti–C vibrations of Ti_3_C_2_T_*x*_. For TiO_2_@C, the Ti–O peak was detected at a frequency of 480 cm^−1^. The presence of adsorbed water molecules on the catalyst surface was observed at 1628 (bending vibrations) and 3550 cm^−1^ (stretching vibrations).^35^ The absence of any Ti–C peaks confirms that all Ti–C–Ti–C–Ti bonds were completely converted into TiO_2_@C, which underwent heterostructures to enhance the activity performances of catalysts during photoreactions.

### Raman analyses

The Raman results for Ti_3_C_2_T_*x*_, Ti_3_C_2_T_*x*_@TiO_2_, and TiO_2_@C were obtained in the 100–800 cm^−1^ region (Fig. 2c). MXenes (Ti_3_C_2_T_*x*_) belong to the D_3d_ point group, and their vibrations can be described using Mulliken symbols as 2(*N* − 2) E_g_ + (*N* − 2) A_1g_ + 2(*N* − 2) E_u_ + (*N* − 2) A_2u_, where *N* represents the number of atoms per unit cell (*i.e.*, five in the case of Ti_3_C_2_).^36^ The E_g_ and E_u_ are doubly degenerate modes (mostly E_g_ is used for representation), while E_g_ and A_1g_ are the only Raman-active vibration modes. In MXene (Ti_3_C_2_T_*x*_), four Raman modes are active: E_g_ and A_1g_ signify the in-plane and out-of-plane vibration of Ti atoms, along with two additional vibration modes of C atoms (Fig. S1†).^37^ The Raman frequency of 208 cm^−1^ (E_g_ mode) is attributed to in-plane vibrations of Ti and C atoms. The carbon layer vibrations appear at higher frequencies (500–800 cm^−1^). The presence of surface terminations (=O, –F, –OH) causes more vibrations due to the increased atoms in the unit cell. The Raman results of Ti_3_C_2_T_*x*_ show three regions: lower frequencies for entire flake vibrations, mid-frequency for surface terminations, and higher frequencies for carbon and surface terminations (Fig. S1†).^36^ The E_g_ corresponds to in-plane vibrations, and A_1g_ corresponds to out-of-plane vibrations. The Raman vibrations around 600 cm^−1^ (E_g_ mode) were attributed to the C–O vibrations. As compared to the pristine Ti_3_C_2_T_*x*_, the Raman peaks of TiO_2_@Ti_3_C_2_T_*x*_ indicate the formation of titanium dioxides, with the vibrations at 147 cm^−1^ that correspond to the anatase phase. Moreover, the Raman spectrum of TiO_2_@C exhibits Raman vibrations at 148 cm^−1^ (E_g_), 397 cm^−1^ (B_1g_), 510 cm^−1^ (A_1g_) and 620 cm^−1^ (E_g_). These vibrations were attributed to the oxygen atoms of O–Ti–O (correspond to the anatase phase).^38^

### TGA analysis

Thermal gravimetric analysis (TGA) of Ti_3_C_2_T_*x*_ in an oxygen atmosphere was conducted to observe changes as the temperature increased up to 900 °C.^39^ The results (Fig. 2d) demonstrate four weight-change steps. The first weight change was due to the loss of water adsorbed on the surface of Ti_3_C_2_T_*x*_. In the second step, the surface terminations and bonded water were released up to 319 °C.^40^ Further increases in temperature led to the oxidation of Ti_3_C_2_T_*x*_, resulting in a subsequent 19.98% increase in mass. The oxidation of Ti_3_C_2_T_*x*_ started at 320 °C and finally began to decompose at 729 °C.^41^ TGA results revealed the thermal stability of Ti_3_C_2_T_*x*_ in an oxygen atmosphere and TiO_2_ formation (oxidation) on its surface.

### SEM studies

**Fig. 3:**
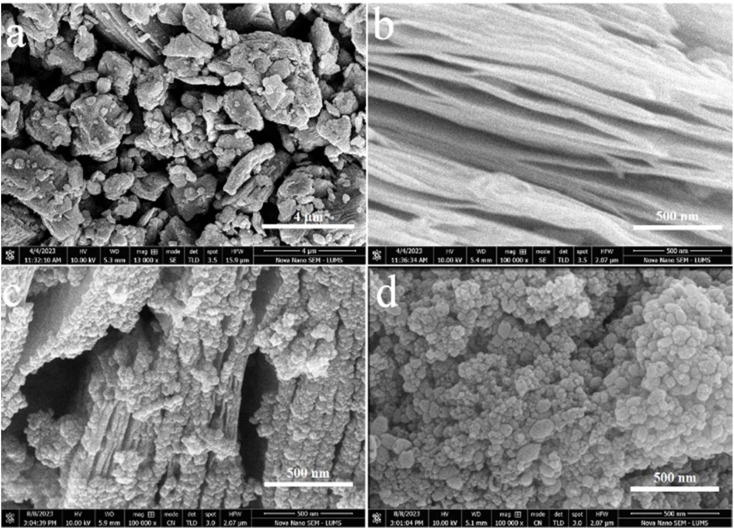
(a) SEM results of MAX (Ti_3_AlC_2_); (b) MXene (Ti_3_C_2_T_*x*_); (c) TiO_2_@Ti_3_C_2_T_*x*_; (d) TiO_2_@C.

Field-emission scanning electron microscopy (FESEM) was utilized to analyse the surface features and characteristics of catalyst.^42^ The SEM results of Ti_3_AlC_2_, Ti_3_C_2_T_*x*_, TiO_2_@Ti_3_C_2_T_*x*_, and TiO_2_@C are demonstrated in Fig. 3a–d. The SEM results of commercial Ti_3_AlC_2_ powder (Fig. 3a) demonstrate the solid block structure with smooth surfaces. The etching of Ti_3_AlC_2_ produces accordion-like multilayer nanosheets loosely bound together,^43^ as shown in Fig. 3b. The MXene layers are clearly separated as compared to the MAX powder that confirms the successful exfoliation (*i.e.*, aluminium removal). The expansive 2D structure of Ti_3_C_2_T_*x*_ nanosheets offers an abundance of active sites and terminations (*i.e.*, –O, –OH, –F) that improve the interactions with reactants for hydrogen generation.^44^ The *in situ* growth of anatase TiO_2_ over the Ti_3_C_2_T_*x*_*via* hydrothermal reactions is revealed in Fig. 3c. The result illustrates the uniform distribution of TiO_2_ particles over the Ti_3_C_2_T_*x*_ surfaces and within the structure of Ti_3_C_2_T_*x*_.^45^ The complete oxidation of MXene produced TiO_2_@C catalysts having uniform dispersion of TiO_2_ across the material. Fig. 3d illustrates a significant difference when compared to pristine anatase (see Fig. S2†). The TiO_2_ derived from Ti_3_C_2_T_*x*_ showcases significantly higher crystallinity compared to other forms of TiO_2_.^46^

### AFM analysis

**Fig. 4:**
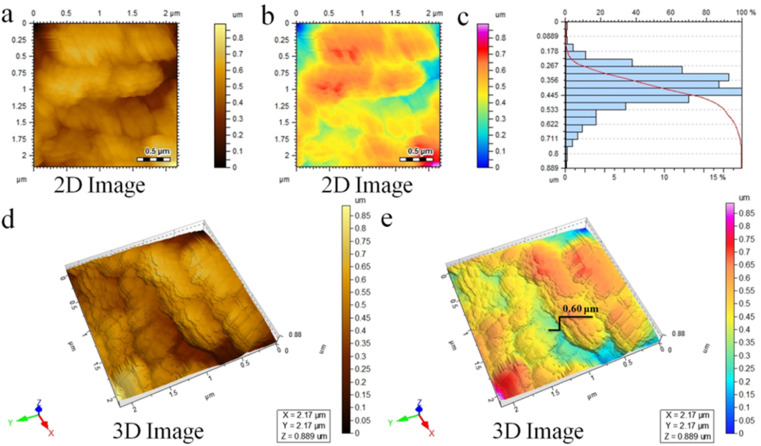
AFM results of the TiO_2_@Ti_3_C_2_T_*x*_ photocatalysts: (a and b) 2D-tapping modes, (c) thickness distribution, and (d and e) 3D images.

Atomic force microscopy (AFM) provides detailed insights into the surface features, morphology, and size distribution of TiO_2_@Ti_3_C_2_T_*x*_ catalysts at nearly atomic scale.^47^ The detailed 2D topographical images are exhibited in Fig. 4a and b. A scan area of 2.17 × 2.17 μm was utilized to assess the height (thickness) distribution within the range of 0.8 μm. The recorded average thickness of 0.445 μm is validated in Fig. 4c. Moreover, Fig. 4d and e reveals three-dimensional results that provide insights to the surface features and roughness of the TiO_2_@Ti_3_C_2_T_*x*_ photocatalysts.^48^

### XPS analyses

**Fig. 5:**
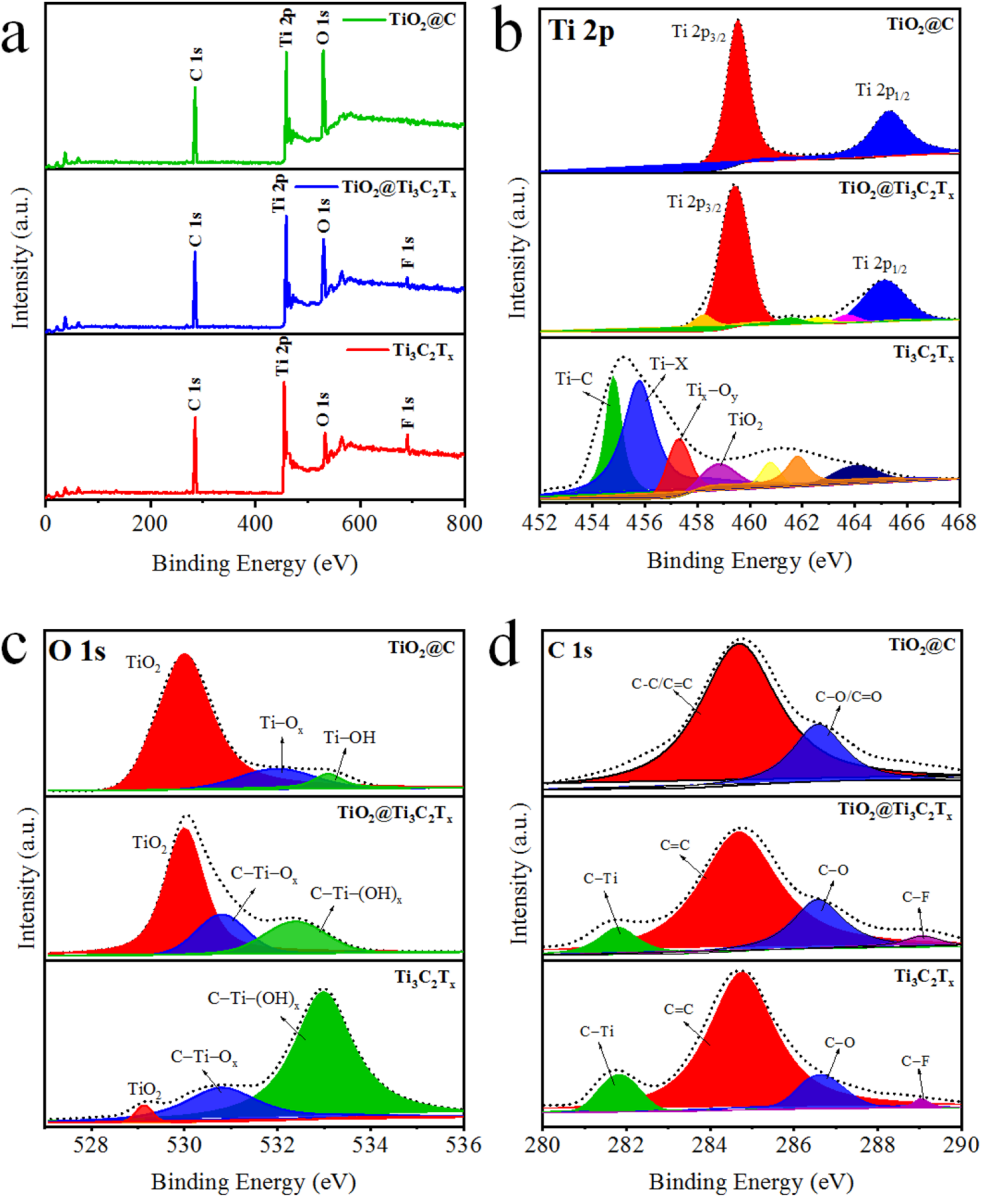
(a) Survey scans, (b) Ti 2p, (C) O 1s, and (d) C 1s XPS results of Ti_3_C_2_T_*x*_, TiO_2_@Ti_3_C_2_T_*x*_, and TiO_2_@C.

The oxidation states and chemical compositions of as-synthesized catalysts were studied by X-ray photoelectron spectroscopy (XPS).^49^ The comparative results (survey scans) of Ti_3_C_2_T_*x*_, TiO_2_@Ti_3_C_2_T_*x*_, and TiO_2_@C are illustrated in Fig. 5a. The XPS results of Ti_3_C_2_T_*x*_ demonstrate the existence of fluorine terminations due to the use of HF during its synthesis.^50^ The binding energies of titanium 2p_3/2_ and 2p_1/2_ were observed at 455.1 and 461.3 eV (see Fig. 5b), which confirms the existence of Ti–C bonds along with various other surface terminations (*i.e.*, =O, –OH).^51^ This spectroscopic study provides a detailed material's composition as well as its surface features. In TiO_2_@Ti_3_C_2_T_*x*_ catalysts, the formation of TiO_2_ over the Ti_3_C_2_T_*x*_ surfaces was confirmed by the sifting of 2p_3/2_ and 2p_1/2_ peaks to 459.3 eV and 465 eV, respectively.^52^ The results depict that complete oxidations of Ti_3_C_2_T_*x*_ cause the removal of surface termination. Note: termination that exists on MXene layered structure. Fig. 5c demonstrates O 1s peaks for the Ti_3_C_2_T_*x*_, TiO_2_@Ti_3_C_2_T_*x*_, and TiO_2_@C catalysts. The Ti_3_C_2_T_*x*_ exhibits C–Ti–(OH)_*x*_, C–Ti–O_*x*_ and TiO_2_ peaks at binding energies of 532.98, 530.78, and 529.18 eV, respectively.^53^ The increased intensity of Ti–O bonds and decreased intensity of C–Ti peaks indicate the *in situ* growth of TiO_2_ on the Ti_3_C_2_T_*x*_ surface. Results depict that oxygen terminal groups were transformed into TiO_2_. The C 1s XPS results demonstrate four peaks fitted at 281.88, 284.88, 286.58, and 289.18 eV, which were assigned to the C–Ti, C=C, C–O, and C–F bonds, respectively (Fig. 5d). The intensities of the C–Ti and C–F peaks decrease in TiO_2_@Ti_3_C_2_T_*x*_ and TiO_2_@C catalysts (as oxidation progresses), which converts the Ti–C bonds into Ti–O bonds.^54^

### UV-vis/DRS and PL studies

**Fig. 6:**
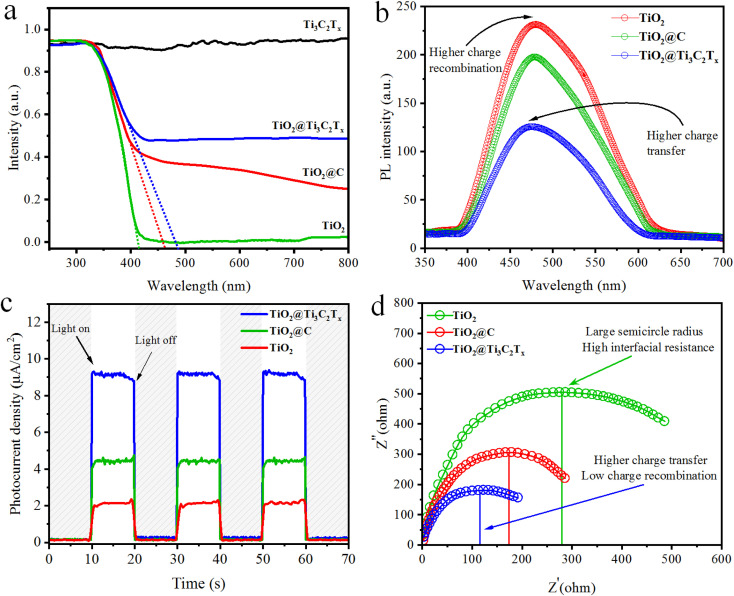
(a) UV-vis/DRS, (b) PL, (c) photocurrent response, and (d) EIS results of TiO_2_, TiO_2_@C, and TiO_2_@Ti_3_C_2_T_*x*_ catalysts.

The optical properties of as-synthesized catalysts were obtained using UV-vis diffuse reflectance absorption spectra as shown in Fig. 6a. The UV-vis/DRS results demonstrate the ability of TiO_2_, TiO_2_@C, TiO_2_@Ti_3_C_2_T_*x*_, and Ti_3_C_2_T_*x*_ to absorb photons, and then bandgap energy values were estimated using the Tauc plot method (Fig. S3†).^55^ TiO_2_ (anatase) displays absorption in the UV region with a bandgap energy of 3.1 eV, whereas Ti_3_C_2_T_*x*_ (MXene) exhibits photon absorption across the entire visible and UV region. The *in situ* growth of TiO_2_ over Ti_3_C_2_T_*x*_ displays an increase in photon absorption.^56^ The increase in light absorption is due to the presence of black-coloured MXene and carbide-derived carbon (CDC).^57^

The charge separation, transfer, and recombination were studied *via* the photoluminescence (PL) technique. The PL intensity of pristine TiO_2_ indicates higher recombination of photoexcited charges (back reaction).^13,58^ The PL intensities of synthesized catalysts suggest that TiO_2_@Ti_3_C_2_T_*x*_ has less charge recombination and can deliver relatively higher H_2_ production; see Fig. 6b. The improvements in electron transport and charge separation are attributed to the development of an *in situ* heterojunction.^59^

### TPC and EIS studies

Electrochemical impedance spectroscopy (EIS) and transient photocurrent (TPC) responses were used to study the charge transfer efficiency. The TPC responses of TiO_2_, TiO_2_@Ti_3_C_2_T_*x*_ and TiO_2_@C were analysed with on/off cycles (Fig. 6c). The results demonstrate that TiO_2_@Ti_3_C_2_T_*x*_ catalysts exhibit a higher photocurrent response as compared to TiO_2_ and TiO_2_@C. The higher photoresponse is attributed to the higher charge separation.^60^

EIS measurements for TiO_2_, TiO_2_@Ti_3_C_2_T_*x*_ and TiO_2_@C catalysts are shown in Fig. 6d. TiO_2_@Ti_3_C_2_T_*x*_ shows a smaller semicircle diameter, which suggests improved interface carrier transfer and decreased interface resistance. TiO_2_@Ti_3_C_2_T_*x*_ catalysts exhibit improved charge separation efficiency and improved charge transfer across interfaces. The lower interface resistance indicates the improved photocatalytic efficiencies.^52^

### BET surface area analyses

**Fig. 7:**
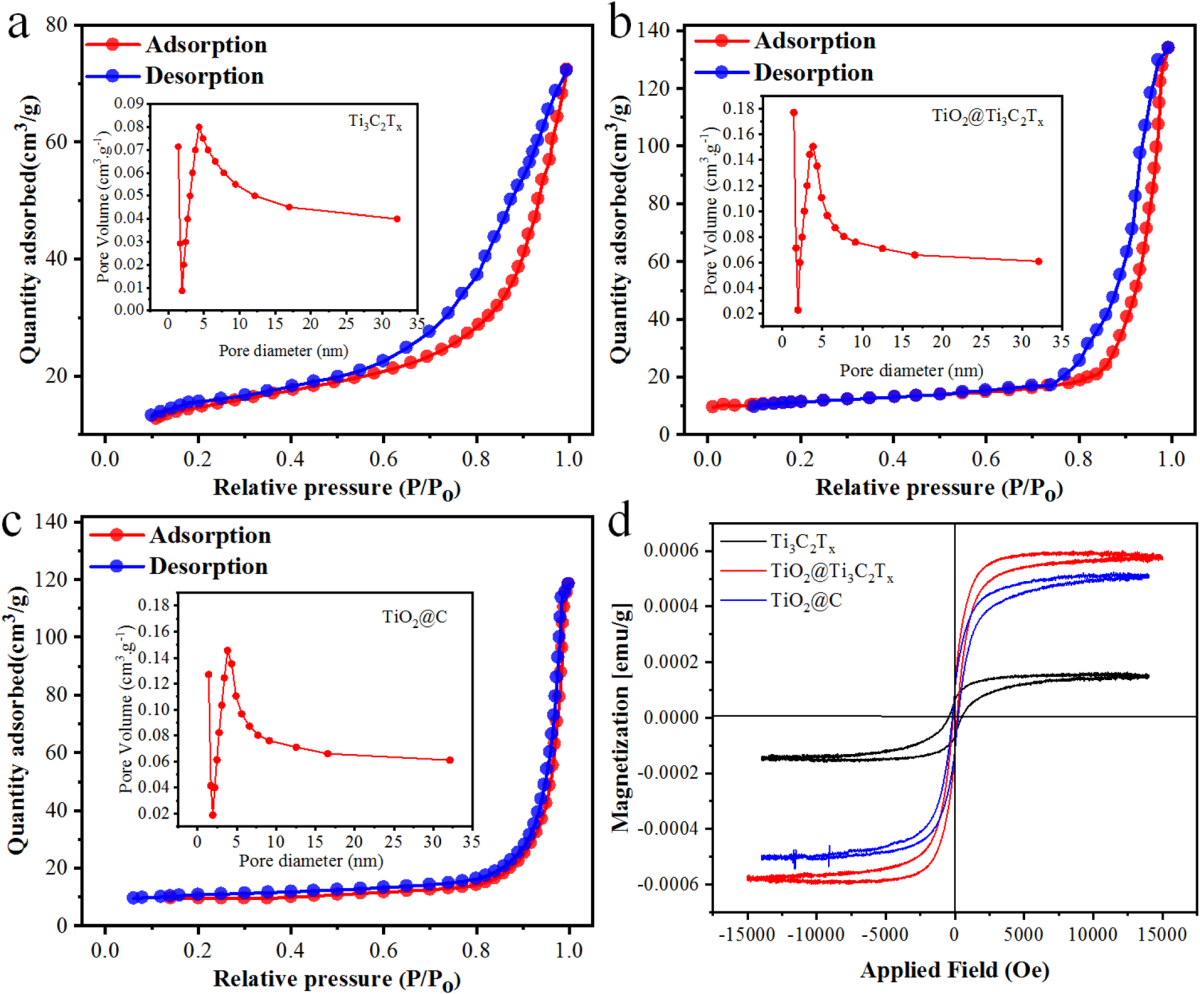
BET results of (a) Ti_3_C_2_T_*x*_, (b) TiO_2_@Ti_3_C_2_T_*x*_, and (c) TiO_2_@C catalysts, and (d) VSM hysteresis loop of Ti_3_C_2_T_*x*_, TiO_2_@Ti_3_C_2_T_*x*_ and TiO_2_@C catalysts.

The surface area of the prepared photocatalysts was determined through nitrogen adsorption–desorption isotherms using BET measurements, as depicted in Fig. 7a–c. All catalysts exhibit typical IV isotherms and H_3_ hysteresis loops according to Brunauer–Deming–Deming–Teller (BDDT) classifications.^61^ The surface areas of Ti_3_C_2_T_*x*_, TiO_2_@Ti_3_C_2_T_*x*_, and TiO_2_@C are 8.12, 32.57 and 28.17 m^2^ g^−1^, respectively. The larger surface area of TiO_2_@Ti_3_C_2_T_*x*_ is due to the *in situ* distributions of TiO_2_ particles over Ti_3_C_2_T_*x*_; hence, the possibility of agglomeration between TiO_2_ particles becomes quite low.^62^ Pore size distributions were calculated and are depicted in Fig. 7a–c, revealing very close pore size distributions with a peak centered at *ca.* 5.0 nm. The TiO_2_@Ti_3_C_2_T_*x*_ and TiO_2_@C catalysts are approximately twice as mesoporous compared to pristine Ti_3_C_2_T_*x*_.^63^ Therefore, the *in situ* growth of TiO_2_ over Ti_3_C_2_T_*x*_ provides a larger and more mesoporous surface area for enhanced H_2_ and O_2_ evolution reactions.

### EPR and VSM studies

The presence and properties of oxygen vacancies were investigated utilizing electron paramagnetic resonance (EPR) spectroscopy.^64^ It is evident from the results (Fig. S4†) that no EPR signal appeared in the pristine Ti_3_C_2_T_*x*_ sample. On the other hand, a distinct signal featuring a G factor of 2.005 was observed in both the TiO_2_@C and TiO_2_@Ti_3_C_2_T_*x*_ catalysts, indicating the trap and transfer of unpaired electrons. Notably, a higher signal was observed in the TiO_2_@Ti_3_C_2_T_*x*_ catalysts as compared to the TiO_2_@C catalysts.^64^ Some recent reports suggest that the existence of oxygen vacancies is more challenging for photocatalytic H_2_ evolution reactions.^65^

The magnetic properties of the as-synthesized catalysts were examined *via* the vibrating-sample magnetometry (VSM) technique (Fig. 7d). This analysis provides crucial insights into saturated magnetic intensity (Ms), residual magnetic intensity (Mr), coercivity (Hc), and squareness ratio.^66^ The TiO_2_@Ti_3_C_2_T_*x*_ and TiO_2_@C photocatalysts exhibit Mr values of 0.064 and 0.071 emu g^−1^ respectively. By using the VSM plots, the magnetic parameters (Ms, Mr, Hc, and squareness ratio) of the as-synthesized catalysts are tabulated in Table S2.†

### Water splitting activities

**Fig. 8:**
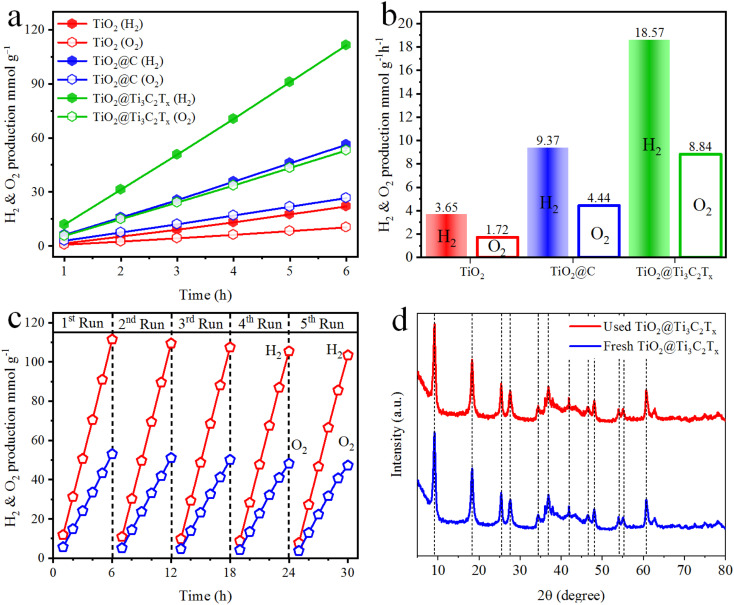
Hydrogen and oxygen production activities of TiO_2_, TiO_2_@C, and TiO_2_@Ti_3_C_2_T_*x*_ (a) mmol g^−1^ and (b) mmol g^−1^ h^−1^; (c) recyclability and (d) XRD results of fresh and used TiO_2_@Ti_3_C_2_T_*x*_ catalysts.

The hydrogen and oxygen evolution rates have been measured to evaluate the catalytic performances. Fig. 8a and b indicate the activities in mmol g^−1^ and mmol g^−1^ h^−1^, respectively. The results demonstrate that H_2_ and O_2_ generation increases linearly with the irradiation time for each photocatalytic reaction. It has been observed that TiO_2_@C and TiO_2_@Ti_3_C_2_T_*x*_ catalysts exhibit 9.37 and 18.57 mmol g^−1^ h^−1^ of hydrogen, respectively. The O_2_ production rate was approximately half that of the H_2_ production rate. It is worth mentioning that the activity in the case of TiO_2_@C is two times higher, while for TiO_2_@Ti_3_C_2_T_*x*_, it is five times higher than pristine TiO_2_. The comparison of H_2_ and O_2_ evolution rates of TiO_2_, TiO_2_@C and TiO_2_@Ti_3_C_2_T_*x*_ catalysts and their %QE are tabulated in Table S3.† The higher catalytic performances of TiO_2_@Ti_3_C_2_T_*x*_ are attributed to the existence of multilayer structures that contribute relatively higher active sites to promote water reduction reactions. Additionally, during the photoreaction, the formation of heterojunctions and Schottky barriers at the interfaces enhances the charge transfer to promote and drag water splitting reactions.^67^ Additionally, the higher surface area also has a considerable impact on the photocatalytic activity. In TiO_2_@C catalysts, carbide-derived carbon (CDC) serves as an electron acceptor and drags charges to improve H_2_ and O_2_ production rates.^68^ Comparatively, TiO_2_@Ti_3_C_2_T_*x*_ is a promising catalyst for water splitting reactions due to its layered structures, higher charge transfer, and large number of active sites available for redox reactions.

Photocatalyst stability and its reusability are crucial factors in accessing the catalyst's practicability. The recycling studies were carried out for five consecutive cycles using TiO_2_@Ti_3_C_2_T_*x*_ catalysts. It is noteworthy that the photocatalytic activity remained steady even after five cycles (Fig. 8c). The slight decrease in activity was caused by catalyst deposition on the inner surface of the reactor wall.^35^ The XRD analysis of both fresh and used TiO_2_@Ti_3_C_2_T_*x*_ catalysts was compared, and it was found that there was no significant difference in crystallinity, as shown in Fig. 8d. These findings suggest that TiO_2_@Ti_3_C_2_T_*x*_ catalysts have exceptional stability, which is due to the *in situ*-grown TiO_2_ over the MXene structures.^69^

### Charge transfer and water splitting mechanisms

**Fig. 9:**
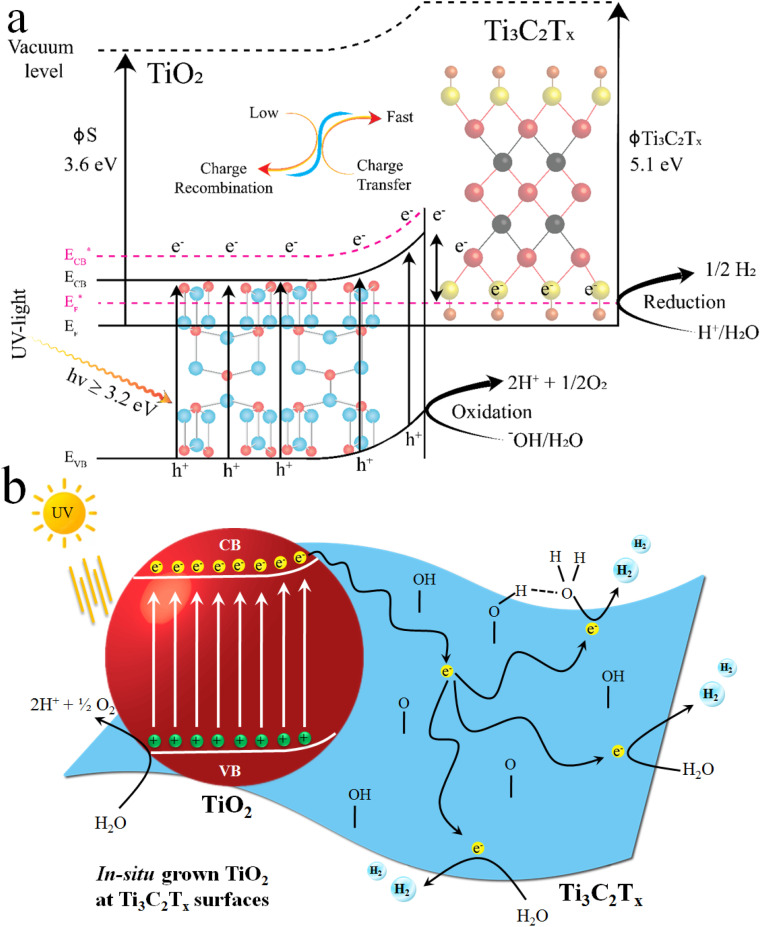
(a) Charge transfer on TiO_2_@Ti_3_C_2_T_*x*_ catalysts; (b) mechanism of water splitting over TiO_2_@Ti_3_C_2_T_*x*_ catalysts.

In a photocatalytic reaction, photons have greater energy than the band gaps, *i.e.*, ≥ *E*_G_, when the excitation of charges occurs on the surfaces of semiconductors. During excitation, electrons start to move from the valence band (VB) to the conduction band (CB) of the semiconductor, leaving behind the electrons deficient charges, *i.e.*, holes.^70^Fig. 9a demonstrates the water splitting process during the photocatalytic reaction. It has been found that TiO_2_@Ti_3_C_2_T_*x*_ exhibits higher catalytic efficiencies relative to the TiO_2_@C catalysts. Higher H_2_ and O_2_ evolution efficiencies in the case of TiO_2_@Ti_3_C_2_T_*x*_ are attributed to the presence of electron promoters, *i.e.*, MXene. It is obvious that MXene's exceptional conductivity contributes to the transportation of electrons to active sites, where a water reduction reaction occurs to generate hydrogen.^71^ Additionally, due to the layered structure of MXene, electron trapping and transfer become relatively high, where electrons can stay on redox sites for further utilization/consumption.^18^ During the photoreaction, a dynamic equilibrium is established between MXene and TiO_2_, which aligns the Fermi energy (*E*_F_) of the TiO_2_@Ti_3_C_2_T_*x*_ photocatalyst to develop a new quasi-Fermi state, 
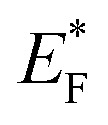
.^72^ Moreover, due to the higher work function of MXene (Ti_3_C_2_T_*x*_), the feasibility of electron population on the TiO_2_@Ti_3_C_2_T_*x*_ system increases, which is why these catalysts contribute relatively higher rates of hydrogen evolution in photoreaction.^73^ MXene develops heterojunctions with TiO_2_; hence, it enhances the feasibility to quench and transfer the charges during photoreaction. That is why TiO_2_@Ti_3_C_2_T_*x*_ effectively promotes electron transfer to the 
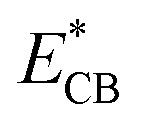
 of TiO_2_.^74^ It is worth mentioning that due to heterojunctions, the Schottky barrier at the interface suppressed the charge recombination (*i.e.*, back reaction).

On the other hand, during catalytic water splitting, oxygen-carrying moieties, *i.e.*, ^−^OH, or peroxide ^−^O_2_, undergo oxidation with holes.^75^ During photoreactions, MXene layers favour the generation of O vacancies when they become exposed to or come into contact with water.^76^ The hydrogen evolution process over TiO_2_@Ti_3_C_2_T_*x*_ catalysts is initiated with the protonation of –O terminations during photoreaction (photon absorptions). It is worth mentioning that in photoreaction protons inherently adsorb on the –O sites instead of Ti sites to *in situ* develops the TiO_2_@Ti_3_C_2_(OH)_*x*_ structures (see eqn (3)–(5)). Subsequently, the adjacent Ti site undergoes protonation. This protonation then favours the enhancement and promotion of catalytic hydrogen.^77^ The catalytic reaction is demonstrated in eqn (3)–(5).

3 Ti–O–Ti + H^+^ + e^−^ → Ti–OH*–Ti

4 Ti–OH*–Ti + H^+^ + e^−^ → Ti–OH*–Ti–H*

5 Ti–OH*–Ti–H* → Ti–O–Ti + H_2_

These above chemical reactions prove the process of protonation and charge transfer involved in hydrogen generation.^77^Fig. 9b illustrates the photoreaction and catalytic hydrogen and oxygen generation over TiO_2_@Ti_3_C_2_T_*x*_ catalysts. In the case of TiO_2_@C catalysts (see Fig. S5†), photogenerated electrons are quenched by the CDC layers to drag the water splitting reactions.^68^

## Conclusions

TiO_2_@Ti_3_C_2_T_*x*_ catalysts have been successfully synthesised and employed for water splitting reactions. In this project, an ethanol-assisted solvothermal approach was introduced to achieve the required morphologies and catalytic characteristics. Typically, TiO_2_@Ti_3_C_2_T_*x*_ and TiO_2_@C catalysts were optimized and assessed for comparative activities. The morphology and optical characteristics were assessed *via* XRD, FTIR, Raman spectroscopy, TGA, SEM, AFM, UV-vis/DRS, XPS, PL, TPC, EIS, BET, EPR, and VSM. The results confirm the morphology, excellent optical response, and charge transfer that indicate the speciality of the current work. The results show that TiO_2_@Ti_3_C_2_T_*x*_ exhibits higher catalytic activity, *i.e.*, 18.57 mmol g^−1^ h^−1^ of H_2_ and 8.84 mmol g^−1^ h^−1^ of O_2_, which is almost double the activity of TiO_2_@C. It is worth mentioning that the higher activity of TiO_2_@Ti_3_C_2_T_*x*_ is attributed to the existence of titania on MXene multilayers that offer more reactive sites and redox centres. The results indicate that heterojunctions between titania and Ti_3_C_2_T_*x*_ executed the rectification and charge transfer to active sites (*i.e.*, redox centres). Additionally, heterojunctions rectify and reduce the back flow of charges during the photoreaction. This significant factor anticipates the consequences of higher catalytic efficiencies. Based on results and activities, it has been anticipated that the current study holds promise to replace costly and conventional catalysts. Although there are many challenges ahead, the described approach has great potential to deliver sustainable hydrogen for the proper implementation of green technologies.

## Conflicts of interest

The authors declare no competing financial interest.

## Supplementary Material

NA-006-D4NA00754A-s001

## Data Availability

The data and necessary protocols of this study have been included as part of the ESI.†
