# Supplementary material for: Unveiling the potential of MXene-fabricated catalysts: an effective approach for H2 generation from water splitting
Source: Nanoscale Adv. 2024 Oct 16;6(23):5861–73. doi: 10.1039/d4na00754a (PMC11523837; doi:10.1039/d4na00754a)
Supplement: NA-006-D4NA00754A-s001 [file NA-006-D4NA00754A-s001.pdf]

Supplementary Information (SI) for Nanoscale Advances.
This journal is © The Royal Society of Chemistry 2024

Supporting Information

Unveiling the potential of MXene fabricated catalysts: An effective approach for H_2 generation from water splitting†

Muhammad Zeeshan Abid[a], *Khezina Rafiq*[a]*, *Abdul Rauf*[a], *Ejaz Hussain*[a]*

[a]Institute of Chemistry, Inorganic Materials Laboratory 52S, The Islamia University of Bahawalpur−63100, Pakistan.

Corresponding authors email: ejaz.hussain@iub.edu.pk; khezina.rafiq@iub.edu.pk

Materials: The following chemicals were used during this study: $TiAl_3C_2$ (Foreman Scientific−china). 40% Hydrofluoric acid (MERK) and absolute ethanol (Sigma Aldrich) and distilled water (PAEC−PK).

Characterizations: Various characterization techniques were employed to comprehensively investigate the catalyst properties and features in order to optimize their performance. X−ray diffraction (XRD) analysis was conducted using a Bruker D2−Phaser instrument to investigate the structural characteristics of the synthesized catalysts. The scan was performed in the 2θ range of 5–80° with a scan rate of 2° min^{-1}. Raman results were recorded using a Raman spectrometer RMP–500/JASCO. Fourier transform infrared (FTIR) results were collected in the 400–4000 cm^{-1} range using a Bruker Alpha Platinum infrared spectrometer in ATR mode. To examine the stability and oxidation behavior of MXene Thermogravimetric Analysis (TGA) was performed using a (TG−209) F−Tarsus NETZSCH Germany instrument. These analyses provide insights into the catalyst's atomic arrangements crystallographic properties and stability. To examine the surface morphology FEI–Nova–450 (Nano−SEM) was employed. The atomic force microscope (AFM–5500, Agilent/USA) was employed to investigate the surface topography at nanometer scale. The oxidation states and surface terminations were examined using an X−ray photoelectron spectrometer (Thermo−ESCALAB 250−Xi) coupled with an Al Ka X−ray source (1486.6 eV). The Optical properties and bandgap were analyzed through diffuse reflectance spectroscopy using UV–2550/Shimadzu. Photoluminescence (PL) spectra were obtained using an LS–45 Perkin Elmer spectrometer. Electrochemical impedance spectroscopy (EIS) analysis was performed using a Solartron impedance analyzer–1260. Photoresponse were monitored using a conventional three–electrode system (λ > 420 nm). Specific surface areas and pore

volumes of the catalysts were determined by Micromeritics Tristar/II−3020 analyzer based on the Brunauer–Emmett–Teller method.

Table S1: The XRD parameters (2θ and corresponding *hkl* values) of Ti_3AlC_2, $Ti_3C_2T_x$, $TiO_2@Ti_3C_2T_x$ and $TiO_2@C$.

**Ti_3AlC_2**		**$Ti_3C_2T_x$**		**$TiO_2@Ti_3C_2T_x$**		**$TiO_2@C$**	
2θ	hkl	2θ	hkl	2θ	hkl	2θ	hkl
9.52	002	9.37	002	9.19	002	25.52	101
19.06	004	18.51	006	18.30	006	37.12	103
33.99	101	27.88	008	25.40	101	38.08	004
36.74	103	60.92	110	27.57	008	48.20	200
39.04	104			37.81	004	54.13	105
41.82	105			48.15	200	55.14	211
48.38	107			53.98	105	62.75	204
56.30	109			55.07	211	69.04	116
60.26	110			60.68	110	70.23	220
				62.79	204	75.11	215

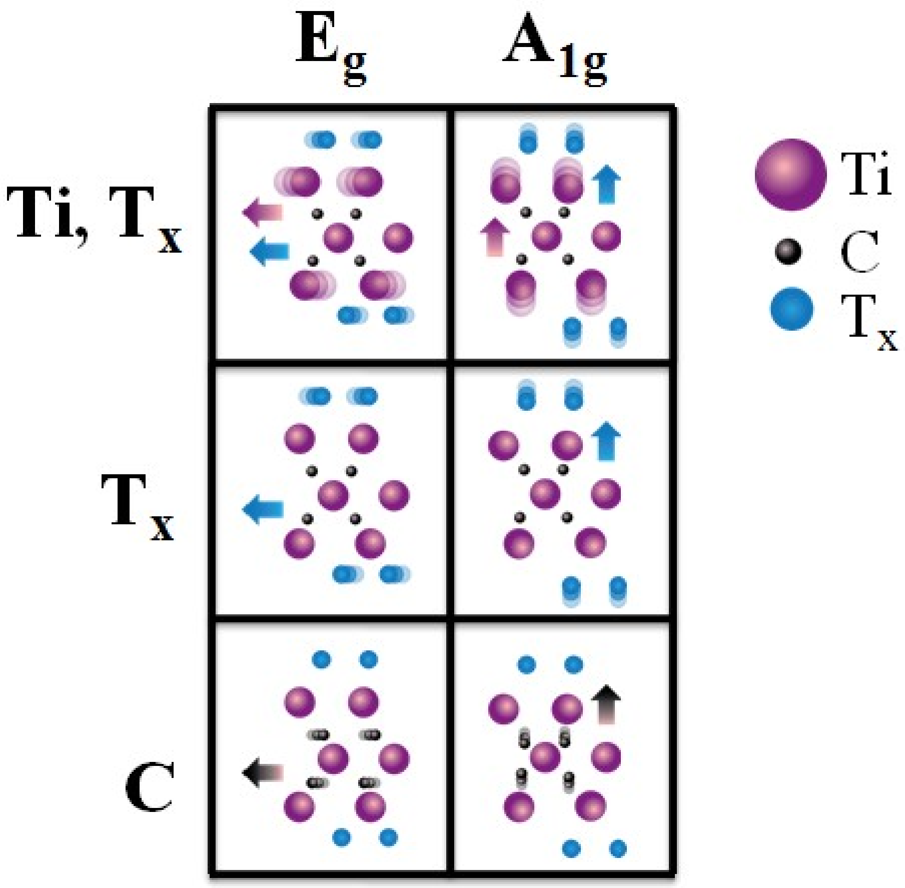

Figure S1: Raman vibrations modes in $Ti_3C_2T_x$.

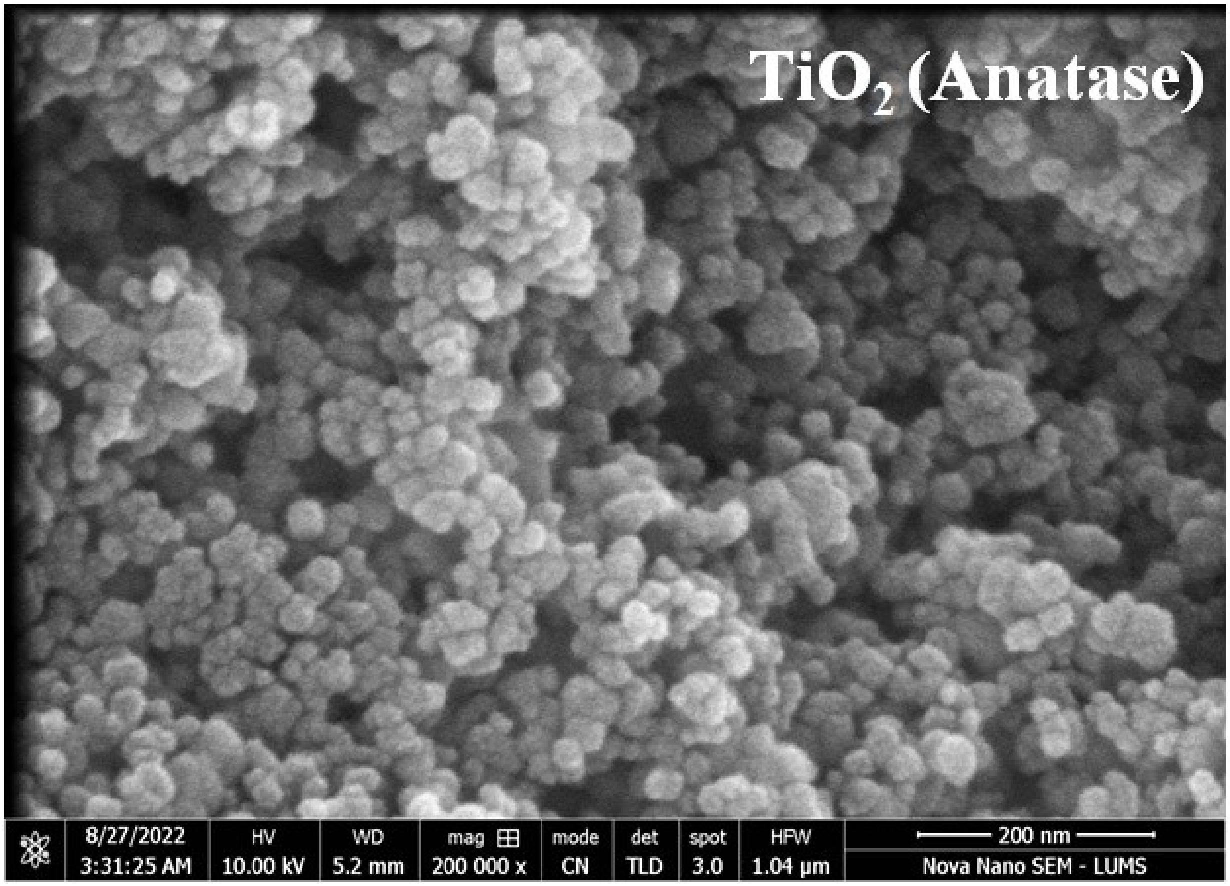

Figure S2: SEM results of pristine TiO_2 at 200 nm.

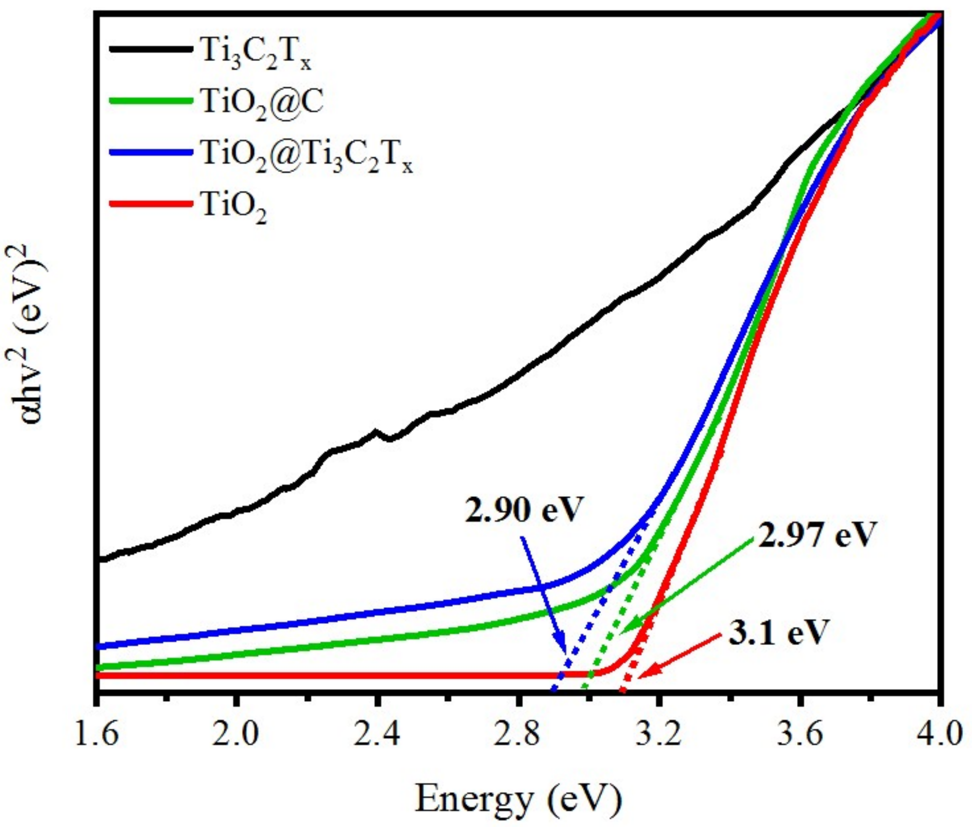

Figure S3: The Tauc plot for bandgap energies of TiO_2, $TiO_2@Ti_3C_2T_x$ and $TiO_2@C$ catalysts.

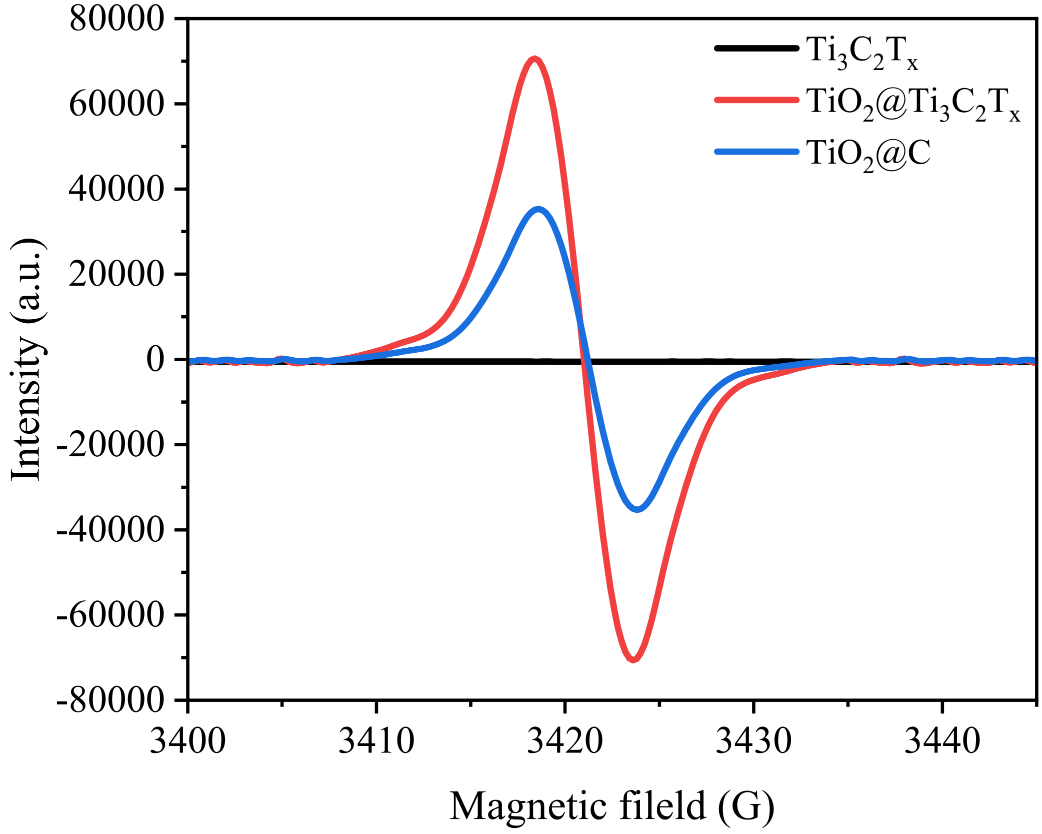

Figure S4: EPR results of $Ti_3C_2T_x$, $TiO_2@Ti_3C_2T_x$ and $TiO_2@C$ catalysts.

Table S2: Magnetic parameters Magnetic saturation, Residual magnetic intensity, Coercivity and Squareness ratio of as-synthesized catalysts.

**Magnetic Parameters**	**$Ti_3C_2T_x$**	**TiO_2@C**	**TiO_2@$Ti_3C_2T_x$**
Magnetic saturation (MS) (emu/g)	0.076	0.291	0.241
Residual magnetic intensity (Mr) (emu/g)	0.030	0.071	0.064
Coercivity (Hc) (G)	518	127	271
Squareness ratio	0.394	0.2439	0.265

Table S3: The comparison of H_2 evolution rate TiO_2, TiO_2@C and TiO_2@$Ti_3C_2T_x$ catalysts.

**Sr. No.**	**Photocatalyst**	**H_2 (mmol g^{-1})**	**H_2 (μmol h^{-1})**	**H_2 (mmol $g^{-1}h^{-1}$)**	**AQY (%)**
**1**	TiO_2	21.95	73.00	3.65	0.9
**2**	TiO_2@C	56.23	187.4	9.37	2.56
**3**	TiO_2@$Ti_3C_2T_x$	111.43	371.4	18.57	5.67

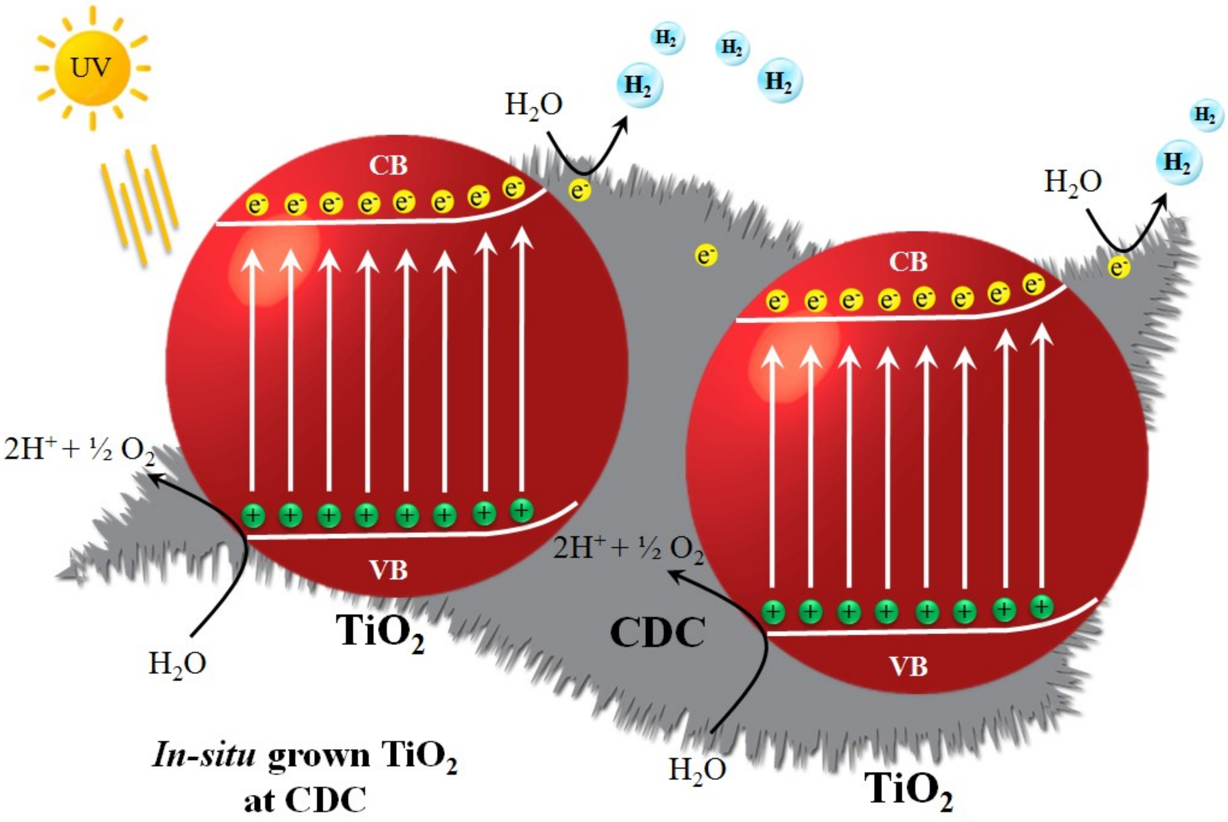

Figure S5: Carbide derived carbon (CDC) layer facilitate the water splitting reaction on TiO_2.
